# P-511. Missed Opportunities for Pre-Exposure Prophylaxis Initiation in Hospitalized Persons with Opioid Use Disorder and Infectious Diseases

**DOI:** 10.1093/ofid/ofae631.710

**Published:** 2025-01-29

**Authors:** Kaley Parchinski, Victor Neirinckx, Angela Di Paola, Tracy Ghantous, Adati Tarfa, Sheela Shenoi, Michelle Strong, Prerana J Roth, Frances Levin, Kathleen Brady, Edward Nunes, Cynthia Frank, Alain H Litwin, Sandra Ann Springer

**Affiliations:** Medical College of Georgia, Athens, Georgia; Yale School of Medicine, New Haven, Connecticut; Yale University, New Haven, Connecticut; Yale School of Medicine, New Haven, Connecticut; Yale University, New Haven, Connecticut; Yale University, New Haven, Connecticut; Prisma Health, Greenville, South Carolina; Prisma Health-Upstate, Greenville, South Carolina; New York State Psychiatric Institute/ Columbia University, New YOrk, New York; Medical University of South Carolina, Charleston, South Carolina; Columbia University, New York City, New York; Yale School of Medicine, New Haven, Connecticut; Greenville Health System/Clemson University, Columbia, South Carolina; Yale University, New Haven, Connecticut

## Abstract

**Background:**

Hospitalizations are increasing among persons with opioid use disorder (OUD) secondary to overdose as well as infections. Hospital settings are a reachable moment not only to offer treatment for OUD and co-occurring infections, but also to offer HIV testing and link to antiretroviral therapy to treat and prevent HIV. Pre-exposure prophylaxis (PrEP) prevents HIV acquisition through both sharing injection drug use equipment and condomless sexual intercourse. Unfortunately, few persons who use drugs including those with OUD are offered PrEP. This descriptive analysis sought to examine patients’ knowledge and attitude about PrEP to better understand if PrEP initiation at time of hospital contact could be used as a way to increase PrEP uptake in patients with OUD.

**Figure 1.**
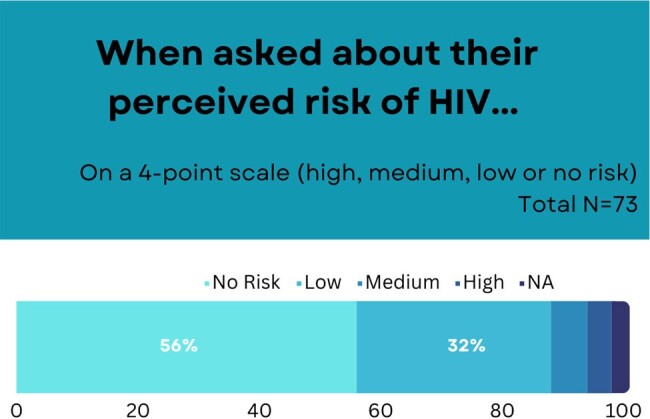
Participants’ perceived risk of HIV on hospital discharge.

**Methods:**

A retrospective data analysis was performed from the ongoing parent study, Project *COMMIT* (Coordinated Medical Treatment of Opioid Use Disorder and Infectious Disease). Adult patients (*N* = 144) hospitalized with bacterial, viral, or fungal infection with moderate to severe OUD were recruited upon admission to hospital and randomized 1:1 to either: 1) infectious disease management of OUD with long-acting injectable buprenorphine vs. 2) treatment as usual. Of the 144, 73 participants who did not report HIV infection, completed a supplemental questionnaire assessing beliefs in PrEP. Basic demographic information, sexual and HIV risk factors, and opioid use risk factors and beliefs about PrEP were reported as frequencies and percentages.

**Results:**

When exploring HIV risk behavior in the past 30 days, 46 (63%) used injection drugs, 26 (35.6%) had condomless sex, 3 (4.1%) knew of their sexual partner being HIV positive or of unknown status, and 12 (16.4%) shared works. Of the 73 who completed the survey, 29 (39.7%) had never heard of PrEP, and none (n=0, 0.0%) had taken PrEP before. When asked about perceived risk of HIV, on a 4-point scale (high, medium, low or no risk), a majority of participants 64 (87.7%) thought they had no or low risk of HIV infection.

**Conclusion:**

This study identified the need for increased education about risk for HIV and PrEP among people who use drugs and attention to acute hospitalization as a reachable moment for engaging them in HIV prevention care.

**Disclosures:**

**Sheela Shenoi, MD MPH**, Merck Pharmaceuticals: Stocks/Bonds (Private Company) **Frances Levin, MD**, Aelis Pharmaceuticals: Grant/Research Support|Indivior: Medication for Research|Major League Baseball: Advisor/Consultant|NCATS: Grant/Research Support|NIDA: Grant/Research Support|NYSPI: Salary Support|SAMSHA: Grant/Research Support|US World Meds: Grant/Research Support **Edward Nunes, MD**, Alkermes: Advisor/Consultant|Alkermes: donated medication or digital therapeutics|Braeburn: donated medication or digital therapeutics|Camurus: Advisor/Consultant|Camurus: donated medication or digital therapeutics|Chess Health: donated medication or digital therapeutics|Indivior: Advisor/Consultant|Indivior: donated medication or digital therapeutics|Pear Therapeutics: Advisor/Consultant|Pear Therapeutics: donated medication or digital therapeutics **Alain H. Litwin, MD, MPH, MS**, Gilead Sciences: Advisor/Consultant|Gilead Sciences: Grant/Research Support|Merck Pharmaceuticals: Advisor/Consultant **Sandra Ann Springer, MD**, Alkermes Inc: Honoraria|Alkermes Inc: In kind study drug donation for NIH sponsored research|Indivior Pharmaceutical company: In kind study drug donation for NIH sponsored research

